# Concordance of Biochip-Based and LC-MS/MS Methods in Urine and Blood Samples in Screening for Amphetamine and Methamphetamine

**DOI:** 10.3390/diagnostics15030269

**Published:** 2025-01-23

**Authors:** Murat Akbaba, Aysun Baransel Isir

**Affiliations:** Department of Forensic Medicine, Faculty of Medicine, Gaziantep University, 27310 Gaziantep, Turkey; aybaransel@yahoo.com

**Keywords:** biochip, amphetamines, methamphetamines, LC-MS/MS, narcotics

## Abstract

**Objectives**: This study aimed to evaluate the efficacy and reliability of LC-MS/MS and biochip-based screening in detecting narcotics from blood and urine samples. **Materials and Methods**: In this single-center study, a total of 250 subjects provided urine and/or blood samples: 234 provided urine and blood samples, and 16 provided only blood samples. Biochip-based narcotics screening was performed on 234 urine and 16 blood samples, and all samples were analyzed using tandem liquid chromatography-mass spectrometry (LC-MS/MS). **Results**: The two most prevalent narcotics were methamphetamine and amphetamine, with both biochip-based array and LC-MS/MS. Cohen’s kappa correlation indicated a substantial agreement between LC-MS/MS and biochip-based screening for both methamphetamine (κ = 0.661, *p* <0.001) and amphetamine (κ = 0.663, *p* < 0.001). LC-MS/MS demonstrated near-perfect agreement between urine and blood samples for both methamphetamine (κ = 0.855, *p* <0.001) and amphetamine (κ = 0.881, *p* < 0.001). **Conclusions**: Biochip-based arrays are a valid method offering a faster, less material-intensive alternative to LC-MS/MS for substance detection in forensic examination.

## 1. Introduction

Narcotic substances have detrimental biological and socioeconomic impacts on users [1,2]. Narcotics-dependent defects in the central nervous system may lead to impairment in perception, logical thinking, attention, and behavior [1,3]. Not all narcotic substances are illegally synthesized: the majority of opioids are prescribed as medications for pain management [4] and amphetamines for attention deficit disorder (ADHD) [5]. However, their misuse outside of treatment categorizes those ‘medications’ as ‘narcotics’ due to the severe decline of one’s mental and physical well-being [4,5,6].

Substance abuse rates are rising globally [7]. The United Nations Office on Drugs and Crime (UNODC) announced that the number of narcotic substance users (aged 15–64) increased from approximately 210 million to 292 million between 2009 and 2022 [8,9]. The global substance abuse rate rose from 4.8% to 5.8%. The 2020 European Drug Report from the European Monitoring Centre for Drugs and Drug Addiction (EMCDDA) estimates that 29% of the population aged 15–64 has used narcotics at some point in their lives [10]. The males and young adults are the risk population for substance abuse [7,9,11]. Turkey has a substantial young population, approximately 13 million adolescent–young adults (aged 15–24), documented by the Turkish Statistical Institute (TÜİK) in 2023 [12]. The young people constitute 15.1% of the Turkish public [12]. While cannabis is the most commonly used drug, cocaine, MDMA (ecstasy), and amphetamines are also seized in large amounts in Turkey [10,11]. Amphetamines are stimulants called ‘uppers’, mainly prescribed to treat conditions such as attention deficit disorder (ADHD) and narcolepsy [5,6]. Methamphetamines are more potent derivatives of amphetamines and are prescribed to treat ADHD and obesity. However, the majority of amphetamine and methamphetamine production is illegal in the form of amphetamine sulfate and distributed worldwide [7,9,13,14].

In order to assure public safety, reliable narcotics testing is essential. Accurate, fast detection of narcotics, as well as subtle newly synthesized derivatives, reduces drug-related accidents, supports legal proceedings, ensures public safety and compliance, and tracks progress during rehabilitation. In forensic investigations, time-sensitive toxicology results can significantly impact case resolution, making rapid biochip assays an attractive option. Thus, with the rise of new psychoactive substances [7,14], golden standards like LC-MS/MS [15,16] and new technologies such as biochip-based screening methods are crucial for accurately identifying substances and preventing false positives [17,18,19,20]. Low concentrations and the traditional derivates of narcotics raise the need for fast, accurate, automated, and high-resolution techniques.

In this study, we aimed to determine the prevalence of narcotic substances, focusing on the context of amphetamines and methamphetamines. We evaluated (a) the reliability and concordance (agreement) between the biochip-based array and LC-MS/MS methods to address the limit of use and (b) whether blood or urine is better for detecting specific narcotic substances.

## 2. Materials and Methods

### 2.1. Study Design and Study Population

**Figure 1:**
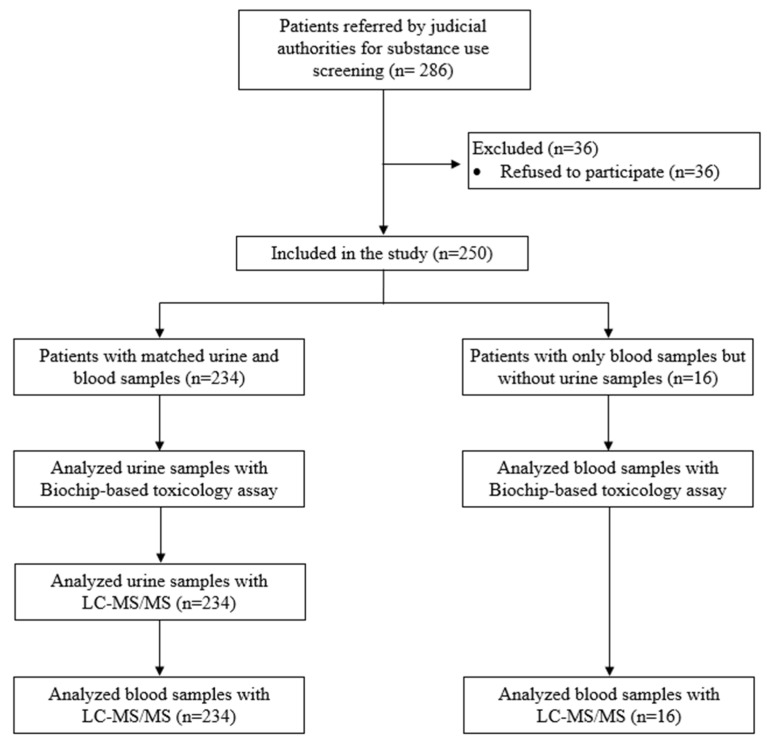
Flow diagram of the study.

This study is a single-center-based study conducted at the Forensic Toxicology Laboratory, Department of Forensic Medicine, Gaziantep University in October 2020. This study included volunteers, and written informed consent was obtained. Research regarding the human subjects was conducted in compliance with the Declaration of Helsinki. Inclusion criteria were as follows: (1) being 18 years of age or over, and (2) being referred to the Department of Forensic Medicine for drug use screening. Those who did not agree to participate in the study were excluded from the study. Two hundred eighty-six subjects were referred to the Forensic Toxicology Laboratory, Department of Forensic Medicine, Gaziantep University, by legal authorities in order to detect narcotics use. Blood and/or urine samples from 250 subjects were collected consecutively (Figure 1). The samples included in the study were (a) both blood and urine samples from 234 subjects and (b) only blood samples from 16 subjects.

### 2.2. Instrumentation

The samples were analyzed with both biochip-based toxicology assay and LC-MS/MS. The LC-MS/MS method is one of the suggested methods for substance detection verification by national regulations. The Triple Quadrupole™ MS 8045 device (Shimadzu Corp., Kyoto, Japan) for LC-MS/MS and Evidence device (Randox Laboratory, Birmingham, UK) for biochip-based toxicology screening were used. For the LC-MS/MS method, the Raptor™ Biphenyl (2.1 × 100 mm, 2.7 µ) column (Restek Corp., Bellefonte, PA, USA) with the Oasis HLB 3cc (60 mg) cartridges (Waters Corp., Milford, MA, USA) for the extraction of blood samples and DTI De-tox TUBE-A (Dyne-Tek Industries, Kansas City, MO, USA) for the extraction of urine samples were used.

### 2.3. Chemicals and Narcotics Standards

Diazepam D5 was used as an internal standard (Chiron, Norway) for LC-MS/MS. Solutions of 5% methanol (*v*/*v*), 70% (*v*/*v*) acetone, and 2% (*v*/*v*) ammonia were prepared for sample extraction with cartridges in LC-MS/MS.

### 2.4. Toxicology Screening

A 0.5 mL amount of each urine sample was processed with the MultiSTAT DOA ToxPlex Urine kit (Randox Laboratory, Birmingham, UK) where 150 µL from each blood sample was processed with the MultiSTAT DOA ToxPlex Blood kit (Randox Laboratory, Birmingham, UK) and loaded onto the biochips according to the manufacturer’s instructions. The samples were run on MultiSTAT DOA ToxPlex Urine Assay program. The array analyzes the following narcotics: methamphetamine (200–1000 ng/mL), MDMA (100–500 ng/mL), amphetamine (200–1000 ng/mL), TCA (150–1000 ng/mL), oxycodone (100–500 ng/mL), opiate (200–2000 ng/mL), 6-MAM (>10 ng/mL), benzodiazepines (100–300 ng/mL or >100 ng/mL), barbiturates (200–300 ng/mL), THC (20–50 ng/mL), dextromethorphan (>20 ng/mL), methadone (200–500 ng/mL), BZG/cocaine (100–300 ng/mL), meprobamate (>500 ng/mL), tramadol (100–300 ng/mL), fentanyl (1–10 ng/mL), buprenorphine (1–10 ng/mL), propoxyphene (>300 ng/mL), PCP (>25 ng/mL), zolpidem (>20 ng/mL), ketamine (100–300 ng/mL), haloperidol (>50 ng/mL), methaqualone (200–300 ng/mL), pregabalin (1000–2000 ng/mL), creatinine (>20 mg/mL), EtG (>1000 ng/mL), acetaminophen (>50 µL), and salicylates (>50 µL).

### 2.5. LC-MS/MS Analysis

The blood and urine samples were extracted as outlined previously [21,22]. Samples were prepared to assess linearity by spiking blood and urine samples (without analyte or internal standard) with standard solutions at concentrations of 1, 2.5, 5, 10, 25, 50, and 100 ng/mL, similar to the previous studies [23,24,25]. For each concentration, 9 independent blood and urine samples were prepared and extracted. A linear graph was plotted based on the relationship between the analyte and internal standard peak areas at each concentration. The linear regression equation and correlation coefficient for the calibration curve were determined. Per validation guidelines, an r^2^ value of ≥0.99 confirms linearity across the tested range. The detected analytes and their detection limits are shown in Table 1.

Five percent methanol solution (*v*/*v*), 70% acetone solution (*v*/*v*), and 2% ammonia solution in ethyl acetate were used in the extraction process for LC-MSMS. Solutions of 2 mm ammonium acetate in 0.1% formic acid (*v*/*v*) (mobile phase A), 0.1% (*v*/*v*) formic acid in methanol (mobile phase B), and 66.6% methanol (mobile phase C) were prepared, and degassed for 10 min, in an ultrasonic bath.

The specifications for the LC-MS/MS setup for this study were as follows: The liquid system flow rate was 0.6 mL/min, the maximum threshold for pressure was set to 660 bar, the injection volume was 10 µL, and the column temperature was 40 °C. Nitrogen and electrospray ionization (ESI+) were used as gas and ion sources, respectively. The dryer gas temperature, dryer gas flow, nebulized gas flow, dissolution temperature, and heater gas flow were set at 300 °C, 10 L/min, 3 L/min, 526 °C, and 10 L/min, respectively. Dynamic multiple reaction imaging (MRM) was used as the scanning mode.

### 2.6. Statistical Analysis

Statistical analysis was conducted using the SPSS for Windows version 23.0 software (IBM Corp., Armonk, NY, USA). Descriptive data were presented as median with interquartile range (IQR) for numeric variables and frequency (*n*) with percentage for categorical variables. Accuracy rates and concordance analyses were used to compare immunoassay and LC-MS/MS methods, as well as urine and blood samples in methamphetamine and amphetamine screening. The accuracy rate was determined by calculating the proportion of cases in which both methods produced either positive or negative results relative to the total number of samples analyzed and was expressed as a percentage (%). Concordance was evaluated using Cohen’s kappa coefficient to assess the agreement between observed concordance and expected concordance by chance. By accounting for chance, the agreement could be compared across different settings and conditions in a way that could not be by an accuracy percentage. Since Cohen’s kappa is affected by prevalence, while the overall accuracy percentage is stable, the incorporation of both values in interpretations of results for health-related research is recommended [26,27]. Kappa results were interpreted as follows: values ≤0 as indicating no agreement, 0.01–0.20 as none to slight, 0.21–0.40 as fair, 0.41–0.60 as moderate, 0.61–0.80 as substantial, and 0.81–1.00 as almost perfect agreement. A *p*-value of <0.05 was considered statistically significant.

## 3. Results

Two hundred and eighty-six adults were referred to the Department of Forensic Medicine to detect narcotics use. Of 286, 36 subjects refused to be involved in the study and hence were excluded from the analysis. The cohort consisted of 250 people; we collected matched blood and urine samples from 234 subjects, and only blood samples from the other 16 subjects. We analyzed urine samples from 234 subjects who also provided blood samples and blood samples from 16 subjects by biochip-based assay. We used LC-MS/MS to analyze matched blood and urine samples (*n* = 234) and only blood samples (*n* = 16). According to the national regulations, if there were differences in pre-screening vs. the verification method in substance detection, the results from LC-MS/MS were accepted as the results of the analysis.

The majority of the samples were collected from males (94.8%) who are young adults (age 24–34) (Table 2).

The biochip-based assay showed that 91% of the urine samples (*n* = 213) and 75% of the blood samples (*n* = 12) were positive for the narcotic substances in the array. Methamphetamine (74%) and amphetamine (70%) were the most prevalent narcotics detected (Table 3).

The analysis of matched blood and urine and only blood samples with LC-MS/MS showed that 95.7% of urine and 88.8% of blood samples contained narcotics. Methamphetamine (81.2%, 81.6%, and 77.2%) and amphetamines (80.8%, 81.2%, and 77.6%) were detected in more than 50% of the overall urine and/or blood samples. The substances following ecgonine methyl ester were only detected in less than 1% of the samples (Table 4).

Using Cohen’s kappa, we determined the concordance between LC-MS/MS and biochip-based narcotic substance screening. The accuracy between the biochip-based assay and LC-MS/MS was 88.4% and 86.7% for methamphetamine and amphetamine screening, respectively. Moreover, there was a substantial agreement between these two methods in both methamphetamine (κ = 0.661 and *p* < 0.001) and amphetamine screening (κ = 0.663 and *p* < 0.001). These values indicate that biochip-based screening reliably detects methamphetamine, with LC-MS/MS serving as a verification method. The strong correlation suggests biochip arrays may efficiently pre-screen narcotic use in forensic cases while maintaining a high degree of accuracy in cross-verification using blood or urine (Table 5).

We focused on the reliability of narcotics detection over urine vs. blood samples using the LC-MS/MS method. Cohen’s kappa coefficient analysis showed a near-perfect agreement between blood and urine samples for methamphetamine (κ = 0.855 and *p* < 0.001) and amphetamine (κ = 0.881 and *p* < 0.001). The accuracy of substance detection exceeded the 95% confidence level for both methamphetamine (95.3%) and amphetamine (96.2%). These findings underline the forensic utility of combining these methods, as the substantial concordance supports using biochip arrays as a rapid and effective screening tool. This helps streamline case processing without sacrificing the accuracy of narcotic detection crucial for legal and rehabilitative decisions (Table 6).

## 4. Discussion

In this study, we evaluated the efficacy and reliability of two methods—LC-MS/MS and biochip-based toxicology screening—in detecting narcotic substances from blood and urine samples. We found that both urine and blood samples were reliable for narcotics substance detection, and biochip-based toxicology screening is a viable alternative for faster analysis. Our cohort was composed of 234 participants contributing both blood and urine samples and 16 subjects contributing only blood samples. The sample pool allowed for a comprehensive analysis of the concordance between urine and blood samples and an assessment of the accuracy of the two detection techniques, LC-MS/MS and biochip-based screening.

The subjects were young adult males (94.8%, age 24–34). According to the World Drug Report 2020, there is a gender-dependent discrepancy among tendencies for substance abuse, where the majority of cases of narcotics users were males in Europe, Egypt, and worldwide [2,9,15,28]. The adults (age 15–64) are the major group to use narcotics, mainly cannabis [9]. The EURO-Den report defines two different age groups in geographically different locations: a younger group (<25 years) in Eastern Europe and an older group (>45 years) in the northern European region, suggesting the impact of socioeconomic/environmental factors on drug use disorder age [29]. In Turkey, the 2020 drug report shows that 91% of the users under treatment were males, and 92.6% were 16–39 years old [11]. In the context of the gender and age of the subjects, the sources of the samples greatly reflect the worldwide risk group.

The World Drug Report states that the major amphetamine market is in the Near and Middle East, and Southwest Asia, whereas methamphetamine (a derivative of amphetamine) is prevalent in North America, and East and Southeast Asia [9,29]. Turkey is one of the regions where a vast amount of fenethylline tablets (an amphetamine-based substance) was seized [11]. Consistent with the seizure numbers and drug reports, our findings demonstrate a high detection rate for methamphetamine and amphetamine across both methods. We detected narcotics in 91% of the urine samples and 75% of the blood samples. LC-MS/MS analysis aligned with biochip-based screening results, showing that 95.7% of urine samples and 88.8% of blood samples contained methamphetamine and amphetamine. These results complement the broader epidemiological trends of methamphetamine and amphetamine use in the Turkish adult population, especially in forensic cases.

Biochip-based screening is a chemiluminescent-based method [19] detecting cytokines [30], toxins [31], antihelminth drugs [32], and drugs of abuse at autopsy [18], in urine [17], and in blood [33]. We analyzed both urine and blood samples on the biochip-based assay and LC-MS/MS and found a substantial agreement between the two methods for both methamphetamine and amphetamine detection (κ = 0.661 and κ = 0.663, respectively, and *p* < 0.001). These findings support the accuracy and reliability of biochip-based assays as a viable alternative to the more established LC-MS/MS method in forensic toxicology settings.

Narcotics testing is mainly associated with criminal cases; thus, it is crucial to get reliable results. The LC-MS/MS method is a gold standard due to its high-resolution and reliable analysis. However, in order to eliminate any false-positive results, the collection of urine samples in conjunction with other biological samples such as hair, oral fluid, and blood is recommended to have more comprehensive and reliable results [34,35,36]. This was due to the fact that substance-derived metabolite concentration is easily affected by (a) the half-life of the metabolite, (b) the hydration level, and (c) the metabolism of a person [34]. Lower water intake may result in more concentrated substance levels whereas greater water intake dilutes the sample blood or urine and might indirectly reduce the concentration of the narcotic substances, even below detection levels. Additionally, the rates at which these substances are metabolized might change from one person to another. If one has a fast-paced metabolism, the substances that were monitored by the forensic toxicology test were broken up faster and lost by the time of detection, which might have hindered their detection. We previously tested the reliability of an alternative method regardless of the sample type and then wanted to confirm whether having an additional biological sample would affect the reliability of the test itself. We used Cohen’s kappa correlation and determined the concordance between matching blood and urine samples in LC-MS/MS analysis. Our findings showed a near-perfect concordance between blood and urine samples for amphetamine (κ = 0.881 and *p* < 0.001) and methamphetamine (κ = 0.855 and *p* < 0.001), further reinforcing the efficacy of LC-MS/MS in forensic drug screening.

The ease of use, the automation, the use of less biological material, and the inexpensiveness are major benefits of biochip-based assays compared to highly specialized LC-MS/MS, making the biochip-based assay an appealing choice for high-throughput screening scenarios. In addition, simultaneously screening for multiple analytes significantly reduces the need for repetitive analyses. The only disadvantage worth mentioning here is that false positives or false negatives are more prevalent due to detection limits compared to LC-MS/MS, and these require confirmation with another method. Conversely, LC-MS/MS is widely regarded as the gold standard. This system requires significant investment, maintenance, and trained personnel for operation and data interpretation. Therefore, the use of LC-MS/MS is impossible, especially for government-based institutions. While LC-MS/MS remains the gold standard, biochip assays provide a pragmatic alternative in resource-constrained environments where rapid preliminary results are critical.

### Limitations of the Study

This study is based on a single center during a short period, and region-specific patterns like methamphetamine and amphetamine use might not be universal. The sample pool was limited to 250 people and was mainly composed of young males. The referral of subjects who are suspected of drug use introduced a selection bias. This potentially biased, referral-based sampling approach might overestimate the prevalence of substances compared to a randomly selected population. The refusal of participation of subjects constitutes approximately 13% of the initial study population, which may add a considerable bias. The excluded subjects, in that way, might mask some rare drug use habits, influence the detection rates of certain substances, or affect analyses about the reliability of screening and verification methods. The demographic homogeneity limits the generalizability of the findings to broader populations, such as females and older individuals. Given that age and gender can influence drug metabolism, detection rates, and patterns of substance use, the current results may not fully represent drug use patterns or detection reliability across all demographics. Although we provided a comparison between biochip-based array vs. LC-MS/MS, our analysis lacks the concordance between urine and blood sample analysis via biochip-based screening. Additionally, the potential for cross-reactivity or false positives inherent to chip-based assay technologies could affect the accuracy of results in populations with metabolic disorders or cases of multidrug use.

## 5. Conclusions

In conclusion, our findings confirm the global trends where the risk group for narcotics use is young males, and amphetamine-type stimulants are prevalent in the Middle Eastern region, including Turkey. We also determined that narcotics detection from blood and urine samples has a near-perfect agreement, reducing the need for the additional conjuncture of biological samples for the analysis. Our narcotics screening analysis showed that a biochip-based array is an accurate and valid screening method and can be used as a preliminary analysis tool. The study demonstrates the reliability of biochip-based screening as an adjunct or alternative to LC-MS/MS, supporting broader adoption in forensic workflows.

## Figures and Tables

**Table 1:** LOD and LLOQ values for detected substances.

Substance	LOD (ppb)	LLOQ (ppb)
Morphine-6-glucuronide	1.30	3.90
Ecgonine methyl ester	1.89	5.67
Cotinine	0.55	1.65
Caffeine	0.01	0.03
Norbuprenorphine-3-β-d-glucuronide	0.01	0.03
Primidone	0.01	0.03
Felbamate	2.09	6.27
Morphine	5.74	17.22
Benzoylecgonine	0.06	0.18
7-aminoclonazepam	0.92	2.76
10-hydroxycarbamazepine	0.04	0.12
Hydromorphone	0.01	0.03
7-aminoflunitrazepam	0.01	0.03
Topiramate	0.12	0.36
Buprenorphine-3-β-d-glucuronide	0.01	0.03
Lamotrigine	0.24	0.72
Codeine	0.93	2.79
Carbamazepine	0.12	0.36
Flumazenil	0.02	0.06
6-MAM	0.45	1.31
Bromazepam	1.04	3.12
Naltrexone	6.82	20.46
Carisoprodol	0.11	0.33
Hydroxyalprazolam	0.01	0.03
Oxazepam	2.15	6.45
Ephedrine	0.02	0.06
Methamphetamine	3.68	11.04
Amphetamine	0.86	2.58
MDMA	0.06	0.18
THC-COOH	2.46	7.38

**Table 2:** Demographics of the patients.

Characteristics (*n* = 250)	
Age (years), Median (IQR)	28.0 (24.0–34.0)
Gender, *n* (%)	
Male	237 (94.8)
Female	13 (5.2)

IQR: interquartile range.

**Table 3:** Substance-positive rates in biochip-based toxicology screening in urine and blood samples.

Substance	Positive in Urine Samples (*n* = 234), *n* (%)	Positive in Blood Samples (*n* = 16), *n* (%)	Positive in Urine or Blood Samples (*n* = 250), *n* (%)
Any substance	213 (91.0)	12 (75.0)	225 (90.0)
Methamphetamine	176 (75.2)	9 (56.3)	185 (74.0)
Amphetamine	169 (72.2)	6 (37.5)	175 (70.0)
Cannabinoids (THC)	84 (35.9)	4 (25.0)	88 (35.2)
Opiate	26 (11.1)	5 (31.3)	31 (12.4)
MDMA	14 (6.0)	3 (18.8)	17 (6.8)
Benzodiazepines^a^	4 (1.6)	3 (18.8)	7 (2.8)
Benzoylecgonine	3 (1.3)	0 (0.0)	3 (1.2)
Buprenorphine	2 (0.9)	1 (6.3)	3 (1.2)
Tricyclic antidepressants	2 (0.9)	0 (0.0)	2 (0.8)
Barbiturates	1 (0.4)	1 (6.3)	2 (0.8)
Phencyclidine	1 (0.4)	0 (0.0)	1 (0.4)

**Table 4:** Substance-positive rates in LC-MS/MS analysis in urine and blood samples.

Substance	Positive Rate, *n* (%)			Substance	Positive Rate, *n* (%)		
	Urine Samples (*n* = 234)	Blood Samples (*n* = 250)	Urine and/or Blood Samples (*n* = 250)		Urine Samples (*n* = 234)	Blood Samples (*n* = 250)	Urine and/or Blood Samples (*n* = 250)
Any substance	224 (95.7)	222 (88.8)	239 (95.6)	Citalopram	2 (0.9)	2 (0.8)	2 (0.8)
Methamphetamine	191 (81.6)	193 (77.2)	203 (81.2)	Lidocaine	2 (0.9)	2 (0.8)	2 (0.8)
Amphetamine	190 (81.2)	194 (77.6)	202 (80.8)	Benzoylecgonine	2 (0.9)	2 (0.8)	2 (0.8)
THC-COOH	99 (42.3)	78 (31.2)	104 (41.6)	Buprenorphine	2 (0.9)	0 (0.0)	2 (0.8)
Ephedrine	40 (17.1)	18 (7.2)	40 (16.0)	Acetylcodeine	1 (0.4)	0 (0.0)	1 (0.4)
Morphine	21 (9.0)	22 (8.8)	22 (8.8)	Diazepam	1 (0.4)	1 (0.4)	1 (0.4)
MDMA	19 (8.1)	14 (5.6)	20 (8.0)	Nordiazepam	1 (0.4)	1 (0.4)	1 (0.4)
6-MAM	17 (7.3)	3 (1.2)	17 (6.8)	Methcathinone	1 (0.4)	1 (0.4)	1 (0.4)
MDA	6 (2.6)	6 (2.4)	6 (2.4)	Trazadone	1 (0.4)	1 (0.4)	1 (0.4)
Quetiapine	4 (1.7)	5 (2.0)	5 (2.0)	Venlafaxine	1 (0.4)	1 (0.4)	1 (0.4)
Alprazolam	3 (1.3)	4 (1.6)	4 (1.6)	O-desmethylvenlafaxine	1 (0.4)	1 (0.4)	1 (0.4)
Heroin	3 (1.3)	1 (0.4)	3 (1.2)	Hydroxyalprazolam	0 (0.0)	1 (0.4)	1 (0.4)
Ecgonine methyl ester	1 (0.4)	3 (1.2)	3 (1.2)	Risperidone	1 (0.4)	1 (0.4)	1 (0.4)
Hydromorphone	2 (0.9)	2 (0.8)	2 (0.8)	9-Hydroxyrisperidone	1 (0.4)	1 (0.4)	1 (0.4)
Cocaine	2 (0.9)	2 (0.8)	2 (0.8)	5F-APINACA	0 (0.0)	1 (0.4)	1 (0.4)

MDA: methylenedioxyamphetamine, MDMA: 3,4-methylenedioxymethamphetamine—“ecstasy”, TCH-COOH: tetrahydrocannabinol carboxylic, 6-MAM: 6-monoacetylmorphine, B3G: buprenorphine-3-glucuronide.

**Table 5:** Concordance of methamphetamine and amphetamine detection between biochip-based toxicology screening and LC-MS/MS in urine samples.

			LC-MS/MS, *n* (%)			Accuracy (%)	Cohen’s Kappa (κ)	*p*
			Negative	Positive	Total			
Methamphetamine	Biochip-based assay, *n* (%)	Negative	37 (15.8)	21 (9.0)	58 (24.8)	88.4	0.661	<0.001
		Positive	6 (2.6)	170 (72.6)	176 (75.2)			
	Total		43 (18.4)	191 (81.6)	234 (100.0)			
Amphetamine	Biochip-based assay, *n* (%)	Negative	39 (16.7)	26 (11.1)	65 (27.8)	86.7	0.633	<0.001
		Positive	5 (2.1)	164 (70.1)	169 (72.2)			
	Total		44 (18.8)	190 (81.2)	234 (100.0)			

**Table 6:** Concordance of methamphetamine and amphetamine detection between urine and blood samples in LC-MS/MS analysis.

			Blood Samples, *n* (%)			Accuracy (%)	Cohen’s Kappa (κ)	*p*
			Negative	Positive	Total			
Methamphetamine	Urine samples, *n* (%)	Negative	42 (17.9)	1 (0.4)	43 (18.4)	95.3	0.855	<0.001
		Positive	10 (4.3)	181 (77.4)	191 (81.6)			
	Total		52 (22.2)	182 (77.8)	234 (100.0)			
Amphetamine	Urine samples, *n* (%)	Negative	43 (18.4)	1 (0.4)	44 (18.8)	96.2	0.881	<0.001
		Positive	8 (3.4)	182 (77.8)	190 (81.2)			
	Total		44 (18.8)	190 (81.2)	234 (100.0)			

## Data Availability

Data are available upon request to the corresponding author.
